# The Design of 3D-Printed Polylactic Acid–Bioglass Composite Scaffold: A Potential Implant Material for Bone Tissue Engineering

**DOI:** 10.3390/molecules27217214

**Published:** 2022-10-25

**Authors:** Sahar Sultan, Nebu Thomas, Mekha Varghese, Yogesh Dalvi, Shilpa Joy, Stephen Hall, Aji P Mathew

**Affiliations:** 1 Department of Materials and Environmental Chemistry, Stockholm University, 114 19 Stockholm, Sweden; 2 Department of Periodontology, Pushpagiri College of Dental Sciences, Kerala University of Health Sciences [KUHS], Medicity, Perumthuruthy, Tiruvalla 689107, Kerala, India; 3 Pushpagiri Research Center, Pushpagiri Institute of Medical Sciences and Research Centre, Kerala University of Health Sciences [KUHS], Thiruvalla 689101, Kerala, India; 4 Department of Biotechnology, Sri Shakthi Institute of Engineering and Technology, Coimbatore 641062, Tamil Nadu, India; 5 Division of Solid Mechanics, Lund University, 221 00 Lund, Sweden; 6 Lund Institute of Advanced Neutron and X-ray Science, 223 70 Lund, Sweden

**Keywords:** polylactic acid, bioglass, 3D printing, bone scaffold

## Abstract

Bio-based and patient-specific three-dimensional (3D) scaffolds can present next generation strategies for bone tissue engineering (BTE) to treat critical bone size defects. In the present study, a composite filament of poly lactic acid (PLA) and 45S5 bioglass (BG) were used to 3D print scaffolds intended for bone tissue regeneration. The thermally induced phase separation (TIPS) technique was used to produce composite spheres that were extruded into a continuous filament to 3D print a variety of composite scaffolds. These scaffolds were analyzed for their macro- and microstructures, mechanical properties, in vitro cytotoxicity and in vivo biocompatibility. The results show that the BG particles were homogeneously distributed within the PLA matrix and contributed to an 80% increase in the mechanical strength of the scaffolds. The in vitro cytotoxicity analysis of PLA-BG scaffolds using L929 mouse fibroblast cells confirmed their biocompatibility. During the in vivo studies, the population of the cells showed an elevated level of macrophages and active fibroblasts that are involved in collagen extracellular matrix synthesis. This study demonstrates successful processing of PLA-BG 3D-printed composite scaffolds and their potential as an implant material with a tunable pore structure and mechanical properties for regenerative bone tissue engineering.

## 1. Introduction

The self-healing abilities of bone can result in the complete reconstruction of damaged tissue, achieving its original form and functionality. However, if a defect reaches a critical size, namely a defect length >2.5 cm and >50% loss of the circumference of the bone [1,2], then the regenerative capacity of bone tissue is insufficient for self-repair [3]. Several methods of reconstructing bone defects are available, namely autograft, allograft, a demineralized bone matrix (DBM), hydroxyapatite calcium phosphate, autologous bone marrow aspirates, bone morphogenetic proteins, and several other related growth factors such as vascular endothelial growth factor (VEGF) and platelet-derived growth factors (PDGF) [4]. The gold standard for bone reconstruction treatment involves autografts and allografts, but like others, these treatments are not risk-free. The drawbacks of these treatments include the need for additional harvesting procedures, donor site morbidity, immunological rejection, disease transmission, and a lack of osteogenetic properties, which can result in bone absorption, non-union, and failure [5,6].

Bone tissue engineering is a state-of-the-art science for restoring damaged bone by the use of porous three-dimensional (3D) scaffolds to provide the appropriate extracellular environment for the regeneration of bone tissues [7]. The success of the scaffold-mediated bone regeneration depends on the biocompatibility, biodegradability, hydrophilicity, osteogenesis, mechanical properties, and architecture [7,8]. The porosity of the scaffold has a direct impact on the mechanical properties, osteogenesis, and integration with natural bone [8]. No definite pore size can be considered ideal for bone regeneration, as micro-porosity (<20 µm) improves bone growth into the scaffolds by increasing the surface area for protein adsorption, increasing ion solubility, and providing attachment points for osteoblasts and vascularization, while macro-porosity (>50 µm) enhances the osteogenic potential of the scaffold [7]. Pore sizes ranging from 50 to 710 μm are suggested for bone regeneration, and it has been stated that macropores in the range of 150–350 μm are ideal [9]. The porosity and mechanical properties of the natural bone are summarized in Table 1 and depend on age, sex, and tested location [7,8].

Bone can be considered a composite material as it has two components: 65–70% inorganic mineral consisting of hydroxyapatite (HA) and 25–30% organic material composed of glycoproteins, proteoglycans, sialoproteins, and bone “gla” proteins [10]. Bioactive glass is a well-known osteoinductive and osteoconductive material and is considered the gold standard in bone tissue engineering [11]. Sol–gel-derived bioactive glasses have superior bioactivity, homogeneity and degradation rates compared with melt-derived glasses of similar compositions. The higher bioactivity is due to the nanometer-scale textural porosity, which increases the specific surface area by two orders of magnitude compared with a melt-derived glass [12,13]. Moreover, the gene activation, bone regenerative capability with better quality and quantity of bone, and high level of bioactivity are unique only to bioglass when compared with synthetic hydroxyapatite [14]. The other preferred aspect of bioglass is the biological fixation, namely the capability of bonding to both hard and soft tissues, whereas hydroxyapatite binds only to hard tissues and needs an exogenous covering to hold the implants in place [15]. The high performance of bioglass in mimicking bone mineralization is attributed to the release of Ca, P, and Si causing Ca and P precipitation at the surface of the implants, with the consequent deposition of amorphous Ca-P crystals. These crystals finally transform into hydroxycarbonate apatite (HCA) by dehydration and promote new bone formation through osteoblast differentiation [16]. However, limitations due to intrinsic brittleness and flaw sensitivity during processing needs to be addressed to exploit the benefits of BG in tissue engineering.

An additive manufacturing technique, specifically fused deposition modeling (FDM), can do wonders to achieve patient-specific scaffolds with precise control over the micro and macro architecture by using images from computer-aided design (CAD), X-ray, and CT and MRI scans [6]. By using this technique, complex internal structures and variations in scaffold designs can be achieved by controlling the amount of material deposition, printing pattern, and printing direction for each layer. This computer-controlled technique is becoming readily accessible, thus reducing the process expenditures and the possibility of human-generated errors [17]. Moreover, FDM allows the high-throughput printing of scaffolds by achieving a storable intermediate filament material that is fed into a printer, which may be advantageous in comparison with solvent-based approaches to fabricating scaffolds [3]. Polylactic acid (PLA) is a medical-grade, US Food and Drug Administration- and European Medicines Agency-approved semi-crystalline polymer widely used for bone tissue engineering and compactible with 3D printing techniques. PLA is a semi-crystalline polymer with a relative high elastic modulus and low elongation at break, as well as mechanical properties that make it a perfect candidate for bone tissue engineering [5].

The combination of BG and PLA can avoid inflammatory reactions and unfavorable environments for cells by buffering the acidic byproducts of polymers with the basic degradation of BG [7]. Estrada et al. demonstrated the FDM of PLA-BG composite filament in fabricating cylindrical scaffolds, but no further data were reported [18]. Distler et al. presented a detailed study on PLA-BG composite filament used for FDM of 3D scaffolds [3]. The in vitro bioactivity and cytocompatibility studies indicated the beneficial impact of BG inclusion in increasing osteogenic differentiation of human adipose-derived stem cells (pre-osteoblast MC3T3E1 cells) in comparison with PLA. However, a decrease in compressive strength was observed with increasing BG loadings due to improper interface bonding (3 MPa for PLA-5%BG).

In the current study, one goal is to have a well-dispersed BG phase in the PLA matrix and thereby positively impact the interface and the mechanical properties. Thermally induced phase separation (TIPS) has been shown to improve the dispersion of hydrophilic nanofillers in the hydrophobic matrix and also generate dry composites for further melt processing as extrusion [19,20,21,22]. Successful processing of PLA composites using the TIPS method was reported earlier for highly challenging reinforcements, such as nanocellulose, which are difficult to disperse. In our recent study, we developed PLA-HaP composite beads by the TIPS method and used them to produce 3D printable filaments through extrusion [20]. In the present study, we report on the TIPS method followed by FDM to develop 3D-printed PLA-BG composite scaffolds with a tuned pore architecture and mechanical properties suitable for bone regeneration by highlighting the in vitro cytotoxicity and in vivo biocompatibility evaluations.

## 2. Results

### 2.1. Thermally Induced Phase Separation (TIPS)

The outer skin of the PLA sphere consists of a smooth polymer surface covered with pores of 1–5 µm and chevron-like patterns due to the initial freezing of the solvent across the droplet surface. The microporous spheres are well formed and have an internal structure consisting of a highly anisotropic channel-like morphology with an internal ladder-like structure. In the composite spheres, BG particles are homogeneously distributed in the PLA matrix throughout the microsphere. The addition of BG leads to a random microstructure with diminished ladder-like structures in the composite spheres. The composite filament shows improved BG dispersion within the PLA matrix due to mixing and grinding of spheres.

### 2.2. Three-Dimensional (3D) Printing

Various 3D scaffolds have been printed with PLA as well as with PLA-BG composite filament in the form of cylinders, discs, and cubes. PLA and PLA-BG cylindrical or disc samples have a broader pore size distribution of 150–300 µm compared with cubic samples, with much narrower pore size ranges of 360–400 µm (PLA) and 450–500 µm (PLA-BG). The topographic SEM images of PLA and PLA-BG 3D-printed scaffolds show that the inclusion of BG particles introduced surface roughness to the 3D-printed scaffolds. The micro-morphological features of the 3D-printed PLA-BG scaffolds were assessed with the use of µCT. It can be observed that the bioglass is homogeneously distributed in the PLA matrix throughout the scaffold. The cross-section images represent the print quality as well as the dispersion and distribution of the bioglass in the walls of the scaffold.

### 2.3. Mechanical Properties

The study showed that an 80% increase in the compressive strength was observed for the 3D-printed scaffolds with the addition of BG particles. The compressive strengths of the PLA and PLA-BG scaffolds were 8 ± 2 MPa and 14 ± 2 MPa, respectively. The modulus was not affected significantly by the addition of BG (PLA 2.8 ± 0.2 and PLA-BG 2.3 ± 0.2 GPa).

### 2.4. In Vitro Cytotoxicity Study

All the various scaffold extracts of 20%, 40%, 60%, 80%, and 100% exhibited similar cell viabilities to that of the control at each time point. Even at the highest tested concentration of 100%, the PLA-BG scaffold exhibited 95.24% cell viability in comparison to that of the control and 10%DMSO negative control, which were 100% and 21.098% at 72 h, respectively. The cell viabilities associated with 20%, 40%, 60%, 80%, and 100% of the PLA-BG were found to be 98.9%, 97.3%, 95.8%, 95.9%, and 96.7%, respectively, at 72 h (*p* < 0.001). Since the percentage of viable cells was more than 70% compared with the untreated negative control, the in vitro cytotoxicity analysis of the PLA-BG scaffold using L929 mouse fibroblast cells confirmed the cell compatibility.

### 2.5. In Vivo Biocompatibility Study

At day seven post implantation, the tissue surrounding both the PLA and PLA-BG scaffolds elicited signs of acute immune response, which involves the infiltration of tissues by inflammatory cells as polymorphonuclear neutrophils (PMNs) and eosinophils seen within a few days after the in vivo implantation of scaffolds. At the 14th day, there was a difference in both the surrounding tissues and in the cell population within the connective tissue surrounding the PLA and PLA-BG scaffolds. The connective tissue surrounding the scaffolds showed a decreased immune response. In addition, the number of polymorphonuclear neutrophils (PMNs) was significantly reduced toward the 14th day. Finally, at the 21st day post implantation, the immune response apparent at the 7th and 14th days was completely resolved, with the surrounding tissue appearing normal. In fact, the tissue in contact with the PLA-BG scaffold contained the same structures as normal connective tissue.

## 3. Discussion

The major advantage of the TIPS technique is the easy dispersion of various fillers into the primary solution and the formation of monodisperse microspheres [21]. In addition, TIPS processing also allows the complete removal of any residual solvent, the formation of spheres with homogeneous size distribution, and the possibility of repeatable production. The sphere size and internal pore structure can be controlled by tuning the processing parameters, such as the type of polymer or solvent, viscosity of the solution, and droplet size [22]. In the TIPS method, the decrease in thermal energy is used as the driving force to induce phase separation of a homogenous polymer solution or a homogeneous multicomponent system which has been formed at a high temperature. Upon cooling, the system becomes thermodynamically unstable and separates into two distinct phases: polymer-rich and polymer-lean (solvent-rich) phases. The solvent in the polymer-lean phase is subsequently eliminated by extraction, evaporation, or sublimation, leaving porosities behind [23]. Because of these advantages, various combinations of fillers and solutions are possible. PLA alone can be processed through the TIPS technique to produce foams [24] and membranes [25]. PLA can also be used together with other materials such as hydroxyapatite [20] and cellulose nanocrystals [26].

**Scheme 1:**
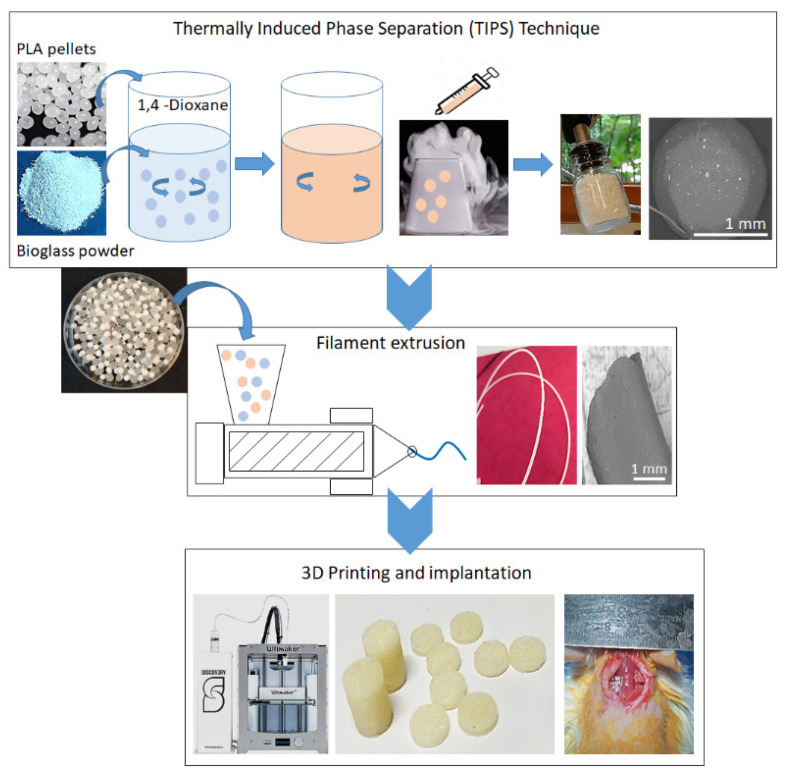
PLA–bioglass scaffold processing and performance evaluation.

In the current study, PLA-BG master batch composite spheres were produced using the TIPS technique, which facilitates the dispersion of BG in the PLA matrix, followed by freeze drying [21]. The freeze-dried composite spheres were extruded into filament that was used to print 3D scaffolds via FDM and were characterized. In vitro cytotoxicity analysis was performed, and thereafter, the scaffolds were implanted into rats to assess their in vivo biocompatibility (Scheme 1).

**Figure 1:**
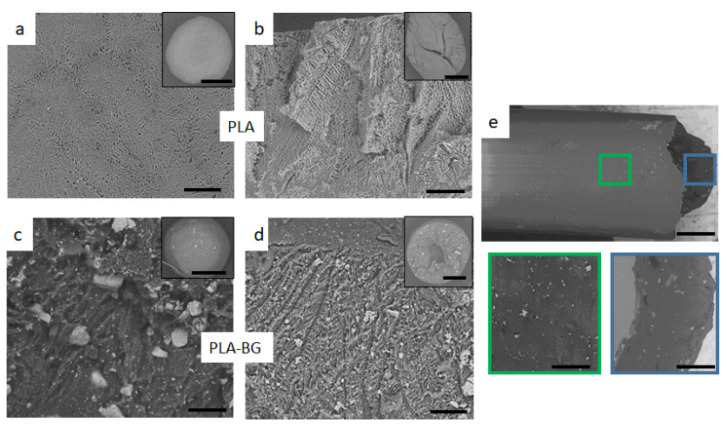
SEM images of freeze-dried TIPS sphere and cross-sections of (**a**,**b**) PLA and (**c**,**d**) PLA-BG. Scale bar = 100 µm, and insect = 1 mm. (**e**) PLA-BG composite filament, showing improved BG dispersion within the PLA matrix, surface (blue square), and cross-section (green square). Scale bar = 50 µm.

Figure 1 shows the SEM of the freeze-dried spheres after TIPS processing (Figure 1a–d). The SEM images of the surface and the cross-section of the PLA and PLA-BG spheres are shown. The outer skin of the PLA sphere (Figure 1a) consists of a smooth polymer surface covered with pores of 1–5 µm and chevron-like patterns due to the initial freeze of the solvent across the droplet surface.

It is known from the literature [21,27,28] that solvent crystallization leads to the internal structure having a highly anisotropic channel-like morphology with an internal ladder-like structure (Figure 1b). This ladder-like structure is a characteristic morphology of foams formed by solid-liquid phase separation of a polymer solution which depends on the cooling rate and the polymer solution [27,28]. Solid-liquid phase separation occurs when the temperature of the polymer solution is lower than the freezing point of the solvent. At this point, the crystallization of the solvent takes place, and the polymer is expelled from the crystallization as impurities forming a polymer-rich phase. After the solvent crystals have been sublimated, pores are formed which are a three-dimensional fingerprint of the geometry of the solvent crystals, giving rise to the outer and inner microstructures [21].

In the composite spheres, BG particles were homogeneously distributed in the PLA matrix throughout the microsphere and were evident even on the exterior surface (Figure 1c). However, the microporous structure became less well-ordered with the addition of BG because of its interference with the crystallizing solvent. These randomly distributed BG particles changed the solvent crystallization by impeding the crystal growth, making the solvent crystals more irregular [27]. In this case, both the polymer and BG particles were expelled during solvent crystallization, so no pattern in the microstructure could be formed, rather having a random microstructure with diminished ladder-like structures (Figure 1d).

Voids or air pockets were evident closer to the center of the microspheres (Figure 1b,d) and were probably formed by entrapment of air during droplet formation. These voids are believed to be prevented by optimizing the processing technique (e.g., using a vibrating needle or adjusting the drops’ distance to the liquid nitrogen) [21]. All the BG particles were bonded to matrix on the surface as well as on the solid walls of the pores. No loose particles were observed during imaging, but there was a broad BG particle size distribution (10–100 µm).

The composite filament was observed under SEM (Figure 1e), which showed further improvement in BG dispersion within the PLA matrix compared with the TIPS composite spheres (Figure 1d). During co-rotating twin-screw extrusion, the material is subjected to high shear forces that can grind particles into smaller sizes [29]. This effect was observed, as the BG particle size decreased significantly after the extrusion process, which narrowed the particle size distribution to 1–15 µm. SEM observation showed that the overall BG distribution improved and the particle size was reduced, though the bioglass remained as clusters.

**Figure 2:**
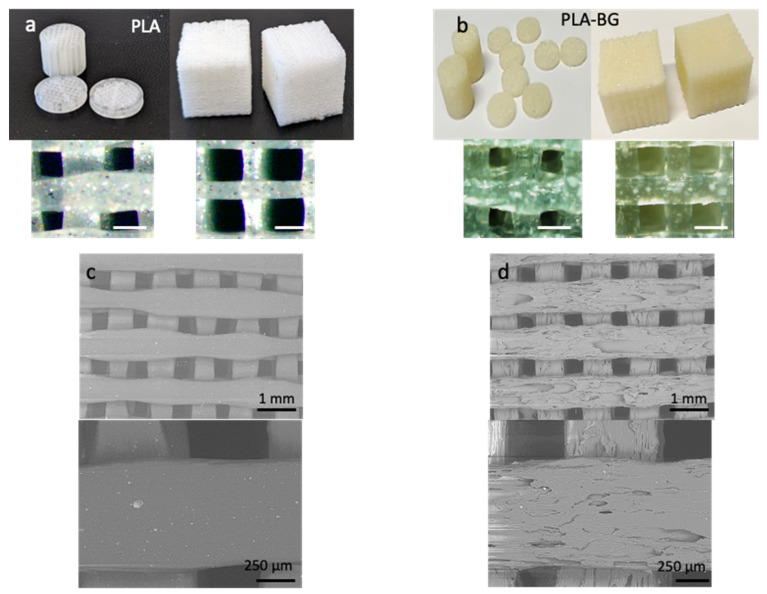
The 3D-printed cylindrical, disc, and cubic scaffolds of (**a**) PLA and (**b**) PLA-BG. Scale bar = 500 µm. Higher magnification SEM images of (**c**) PLA and (**d**) PLA-BG 3D printed cubic scaffolds.

Various 3D scaffolds were printed with PLA (Figure 2a) as well as with PLA-BG composite filament (Figure 2b) in the form of cylinders, discs, and cubes. It can be seen that while printing scaffolds with smaller areas (cylinders and discs with a radius of 6 mm), the printed filament diameter increased due to the slower print speed. To obtain fine details in a small-area print, a smaller nozzle or slower speed was maintained. The fast print speed caused sharp and sudden movements of the print head and made the extruding filament harder to stop, reverse, and start again, causing unsteady movements and reduced print quality. In addition, the circular samples had an increased range of pore size distribution as well as different pore shapes, as the pores closer to the circumference would not be of the same size and shape and would not be perfect squares. Because of these effects, the PLA and PLA-BG cylindrical and disc samples had a broader pore size distribution of 150–300 µm compared with the cubic samples, with much narrower pore size ranges of 360–400 µm (PLA) and 450–500 µm (PLA-BG).

The pore size of the scaffolds was in a desired range for bone tissue engineering. It is widely accepted that the best osteoinduction is achieved with 100–500-μm interconnected pore scaffolds [5]. In addition, a large pore size could improve the infiltration of new blood vessels into the scaffold, enhancing its vascularization and providing cell survival into the inner part of the scaffold [30]. The large pore channels running throughout the scaffolds were considered beneficial in the supply of essential nutrients and the formation of blood vessels [31].

Figure 2 and d shows the SEM topographic images of PLA and PLA-BG 3D-printed scaffolds. It can be seen that the inclusion of BG particles introduced surface roughness to the 3D-printed scaffolds. This is because the nature of the composite filament makes it challenging to have a smooth surface finish, as the effortless flow of the melted PLA is being interrupted by non-melted rigid particles, hence introducing surface features contributing to the nanoscale roughness. Nanoscale roughness is an important aspect of fabricated 3D scaffolds that enhances the functionality in terms of cell behavior. The fibers of the extracellular matrix (10–300 nm in diameter), their interconnecting nanopores, and hydroxyapatite crystals (4 nm) found in natural bone have nanoscaled dimensions [32]. In general, the presence of nanoscale features such as ridges, steps, and grooves increases cell attachment and proliferation, and ridges as thin as 70 nm guide cytoskeletal assembly [33]. Furthermore, the increase in the nanoscale roughness of the scaffold pore walls showed increased cell attachment and proliferation as well as promoting elastin and collagen production [34].

**Figure 3:**
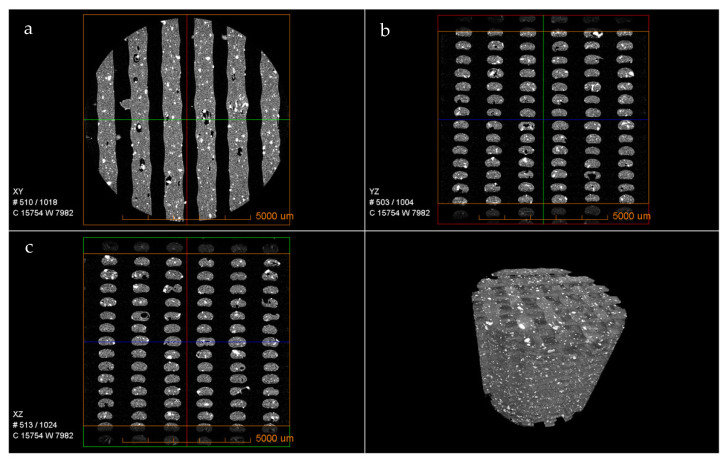
Tomography of the cylindrical 3D-printed PLA-BG scaffold showing the structure along the XY, YZ, and XZ planes and the 3D image.

The micro-morphological features of the 3D-printed PLA-BG scaffolds were assessed with the use of µCT (Figure 3). It can be observed that the bioglass was homogeneously distributed in the PLA matrix throughout the scaffold. The cross-section images (Figure 3a–c) represent the dispersion and distribution of the bioglass in the walls of the scaffold as well as the print quality. Overall, both the dispersion and distribution were consistent and homogenous both in the outer walls and around the pores (see Supplementary Videoes S1 and S2). The 3D structures in Figure 2 as well as the videos in the Supplementary Materials indicate that the dispersed bioglass was largely accessible to the biological environment during the in vitro and in vivo studies.

**Figure 4:**
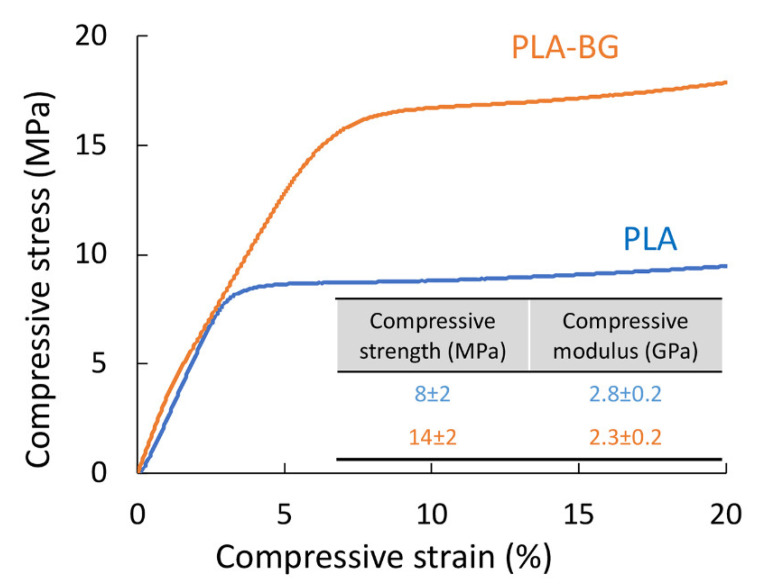
Mechanical data of 3D-printed scaffolds.

The mechanical properties in compression mode are considered relevant for the application of 3D-printed scaffolds for use as materials for bone regeneration. The study showed that an 80% increase in the compressive strength was observed for the 3D-printed scaffolds with the addition of BG particles (Figure 4). In fact, both the PLA and PLA-BG scaffolds had compressive strengths (PLA 8 ± 2 and PLA-BG 14 ± 2 MPa) that lied in the upper range for trabecular bone (2–12 MPa) [7,34]. The modulus was not affected significantly by the addition of BG (PLA 2.8 ± 0.2 and PLA-BG 2.3 ± 0.2 GPa) and fulfilled the requirement for trabecular bone (0.5–20 GPa) [35,36,37]. The improvement in mechanical properties due to the addition of BG was statistically significant.

In our study, we observed an increase in compressive strength when 5% BG particles were added to the PLA matrix. The increase in strength observed in the current work can be attributed to the homogeneous distribution of BG particles within a PLA matrix that was observed in microscopic images of spheres after TIPS processing (Figure 1c,d), in the composite filament (Figure 1e), as well as in the 3D-printed scaffolds (Figure 2). The particle size distribution may also contribute to better mechanical properties, where smaller BG particles may act as filler between spaces left uncovered by bigger particles. It is worth pointing out that in the study by Distler et al., a decrease was observed (0BG 18 ± 10 and 5BG 3 ± 2 MPa) with the addition of a similar amount of BG particles [3]. The reason suggested was improper interfacial adhesion. In addition, much lower modulus values were reported (0BG 0.6 and 5BG 0.5 ± 0.1 GPa) compared with our study (PLA 2.8 ± 0.2 and PLA-BG 2.3 ± 0.2 GPa). In the literature available to date, these are the only available data on mechanical properties for PLA-BG scaffolds printed through the FDM technique. Estrada et al. also reported on 3D scaffolds via FDM of the PLA-BG filament, but no mechanical data were reported [18].

The higher mechanical properties reported using the PLA-BG system were through direct solvent printing with a BG content as high as 50 wt% and using polyethylene glycol (PEG) as a plasticizer. Barbeck et al. reported a bi-layered 3D-printed scaffold combining a PLA layer and a biphasic PLA-BG layer having a compressive modulus around 44.19 ± 2.67 MPa [38]. In the case of Serra et al., PLA and CaP glass were used to fabricate 3D scaffolds with two design patterns: orthogonal and displaced double layers [30]. This work also highlights the importance of the design chosen for the scaffolds to be 3D printed, as an increase in the compressive modulus was observed with the orthogonal design (99.81 ± 3.55 MPa) compared with the displaced layer design (44.19 ± 2.67 MPa) while keeping all the other parameters constant.

**Figure 5:**
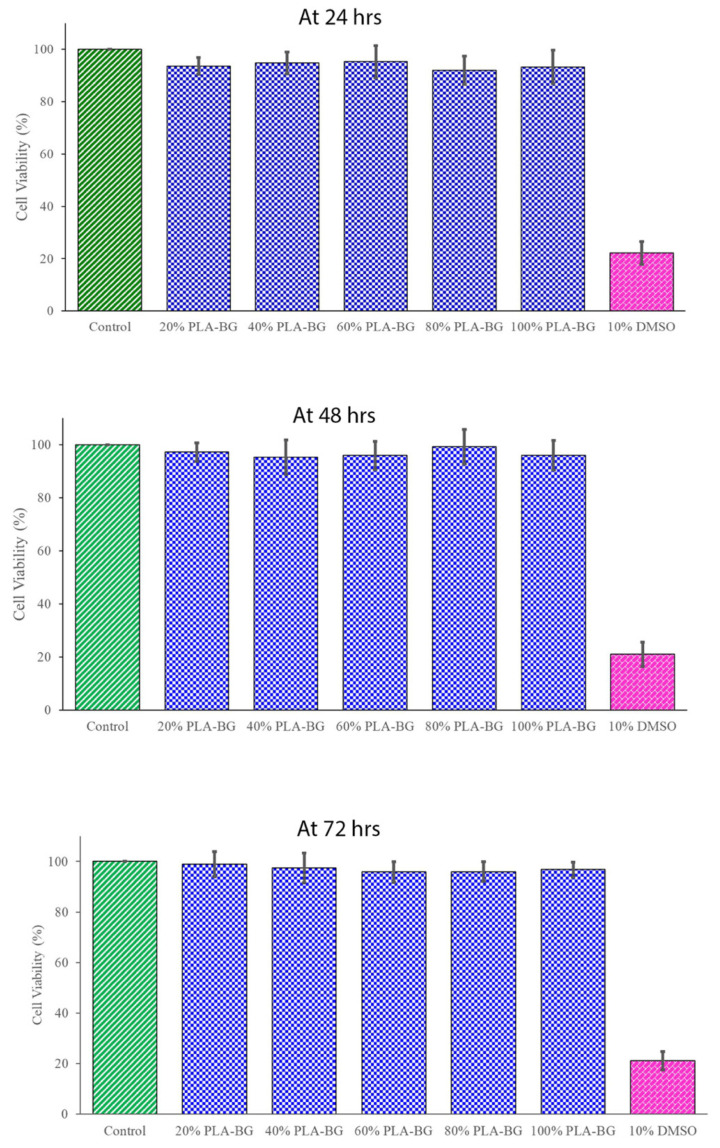
The 3-(4,5-dimethylthyazol-2-yl)-2,5-diphenyltetrazolium bromide (MTT) assay of PLA-BG scaffolds at 24, 48, and 72 h. Cell viabilities associated with 20%, 40%, 60%, 80%, and 100% of the PLA-BG scaffold extract are shown.

The cytotoxicity tests were considered a screening assay for evaluating the living cell reaction to the scaffold in a cell culture assay, which included cell viability and cellular proliferation. The cells were tested utilizing the biomaterial’s extracts from semi-physiological media. In the present study, according to ISO guidelines [39], cytotoxicity analysis using an MTT assay for PLA-BG was performed at 24, 48, and 72 h (Figure 5). The cell viability in the presence of a scaffold was not disturbed and was similar to the control without a scaffold. The difference was not statistically significant, except for the negative control (10% DMSO). Since the percentage of viable cells ware more than 70% compared with the untreated negative control, the PLA-BG scaffold in vitro cytotoxicity analysis using L929 mouse fibroblast cells confirmed the cell compatibility.

**Figure 6:**
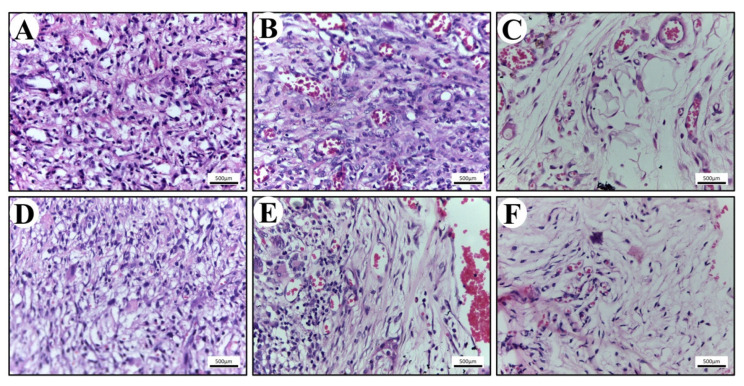
Gross evaluation, hematoxylin, and eosin staining after 2, 4, and 8 weeks post implantation. (**A**–**C**) Gross appearances of the connective tissue surrounding 3D-printed PLA scaffolds. (**D**–**F**) Gross appearances of the connective tissue surrounding 3D-printed PLA-BG scaffolds. Scale bar = 500 µm.

The in vivo biocompatibility of the scaffold was also studied to evaluate the clinical significance of the study. Numerous inflammatory cells were found infiltrating the scaffolds (Figure 6). The population of cells within the connective tissue around scaffolds consisted mainly of granulocytes, specifically polymorphonuclear neutrophils (PMNs) and a few eosinophils. Inflammatory response was seen more in PLA compared with the PLA-BG group. All these tissue reactions are consistent with an expected acute foreign body reaction that follows implantation [40,41,42]. On the 14th day, the population of cells within the epidermis surrounding the scaffolds contained higher levels of macrophages and lymphocytes. Macrophages are known to secrete extracellular matrix proteins and pro-fibrogenic factors, which has an impact on the fibro-proliferation and angiogenesis, resulting in tissue regeneration [42]. This is a characteristic of the foreign body’s reaction to an implanted biomaterial, demonstrating the scaffold cleaning process [40,41,42].

On the 21st day post implantation, the tissue around the perimeter of the PLA and PLA-BG scaffolds showed a lower density of cells due to the decreased inflammation. The population of cells at this stage contained an elevated level of macrophages and active fibroblasts identified through morphological analysis (H&E staining). The active fibroblasts (appearing spindle-shaped) were observed more around the PLA-BG scaffolds, which were actively involved in collagen extracellular matrix synthesis. These observations are in accordance with the previous studies representing the bio-compatible nature of the scaffold with the host [43]. The porous, mechanically efficient, and biocompatible scaffold was proven to be noticeable in utilization for a preclinical approach in hard tissue regeneration. This may have wide therapeutic significance in bone tissue engineering.

## 4. Materials and Methods

The materials included PLA filament with a dimeter of 2.85 mm supplied by Structur3D Printing. PLA colorless pellets were supplied by a filament producer company (Add North 3D AB, Stockholm, Sweden) and used for the production of the PLA-BG composite. The 1–4 dioxane was procured from Sigma Aldrich (Stockholm, Sweden), and 45S5 BG was synthesized via the sol–gel technique with an average particle size of 150 nm, as measured from transmission electron microscopy, that was stable up to 800 °C as reported in our previous studies [44].

For thermally induced phase separation (TIPS), the master batch of PLA-BG composite spheres was prepared using the TIPS technique, where 20 g of PLA pellets were dissolved in 200 mL of 1–4 dioxane (10 wt %) by magnetically stirring at room temperature for 24 h. Then, 15 g of BG was added to this PLA solution after grinding in a ceramic pestle and mortar and being magnetically stirred for 2 h. This composite dispersion (as well as pure PLA in 1–4 dioxane as a control sample) was added to a 20-mL syringe and was manually extruded dropwise into a bath of liquid nitrogen at a distance of 5 cm. To prevent microsphere agglomeration, each droplet was allowed to equilibrate to the liquid nitrogen temperature, denoted by sinking, prior to the addition of further droplets. The droplets solidified upon contact with liquid nitrogen, forming spheres, and they were placed in a freezer overnight. After that, freeze-drying was performed for 24 h.

For filament extrusion, 265 g of pure PLA pellets were mixed into the master batch of PLA-BG spheres, and filaments with 5% BG composition were produced by Add North 3D AB via melt extrusion. A continuous filament 2.85 mm in diameter was prepared for 3D printing.

Regarding the three-dimensional printing materials, PLA-BG composite filament was used to 3D print scaffolds via FDM with a nozzle diameter of 400 μm, nozzle temperature of 210 °C, print bed temperature of 90 °C, flow rate of 100%, and printing speed of 100 mm/s. Two types of porous scaffolds were printed: (1) cylindrical scaffolds (RxL: 6 × 150 mm) and disc samples (RxL: 6 × 3 mm) and (2) cubic scaffolds (20 mm^3^).

As for microscopy, for imaging the composite filament and 3D-printed scaffolds, an optical microscope (Olympus stereomicroscope model SZX12, Tokyo, Japan) equipped with a cross-polarizer was used. Image J was used to measure the particle size. JEOL JSM-7401F (JEOL, Tokyo, Japan) and Hitachi TM3000 (Hitachi, Tokyo, Japan) scanning electron microscopes were used to analyze the surfaces and cross-sectional morphologies of the PLA and PLA-BG composite freeze-dried spheres after TIPS processing. Moreover, SEM observation was used to evaluate the architecture of the 3D-printed disc and cubic composite scaffolds.

Regarding the mechanical properties, Instron 5960 with a 100-N load cell was used for compression testing of the 3D-printed pure PLA and PLA-BG composite cubic scaffolds. The samples were tested at a speed of 1 mm/min without preloading. Stress–strain data were computed from load–displacement measurements. The compressive modulus was determined based on the slope of the stress–strain curve in the elastic region for seven samples.

For in vitro cytotoxicity analysis, a 3-(4,5-dimethylthyazol-2-yl)-2,5-diphenyltetrazolium bromide (MTT) assay was used for the evaluation of cytotoxicity analysis, as reported in an earlier study [44,45]. The mouse L929 fibroblast cell line was seeded at 3 × 10 with 4 cells/mL in a 96-well plate and cultured using DMEM medium. The media were enhanced with 10% fetal bovine serum and 1% penicillin-streptomycin solution. The cells were cultured at 37 °C with a 5% CO_2_ supply overnight before treatment with a scaffold. The scaffolds were cut to 10 mm in size, sterilized using ethylene oxide, and incubated in the cell medium. Various concentrations (20%, 40%, 60%, 80%, and 100%) of 3D-printed PLA-BG-incubated extracts (100 µg/mL) were prepared in DMEM medium, and 10% dimethyl sulfoxide (DMSO) was used as a negative control (ISO 10993-5, 2009) [39] for different timelines (24, 48, and 72 h). For in vivo biocompatibility, adult male Sprague Dawley rats weighing 250–350 g were purchased from Kerala Veterinary College in Mannuthy, India and used for this study. The in vivo experiments were carried out with prior permission from the Institutional Animal Ethics Committee and were conducted while strictly adhering to the guidelines of the CPCSEA, constituted by the Animal Welfare Division of the government of India and the Pushpagiri Institute of Medical Sciences and Research Centre in Tiruvalla, India.

The rats were provided with a standard environment with controlled conditions of 23 ± 5 °C, a 12-h light-dark cycle, standard laboratory animal feed (Krish Scientist’s Shoppe, Bangalore, Karnataka, India), and UV sterile water. On the day of surgery, they were anesthetized by intramuscular injection of 5% xylazine (10 mg/body weight) and 10% ketamine (50 mg/body weight). The hairs at the dorsum of the rats were shaved with a Philips electric pet care trimmer, and the skin was cleansed using a 2% povidone iodine solution. A linear incision 8 mm in length was made on the dorsum of the rats using a number 15 BP blade, and a full-thickness flap was elevated. For the experiment, two groups of animals were selected with same age and same weight. The test group received a PLA-BG composite scaffold, and the control group received a PLA scaffold. Sterile scaffolds were implanted subcutaneously and closed by giving interrupted sutures with number 3 black braided silk. The animals received a subcutaneous injection of 1 mg/kg analgesic butorphanol immediately after surgery and 24 h later to reduce postoperative pain. The sutures were removed after the 7th day and carefully monitored postoperatively.

Regarding scaffold resection, at 7, 14, and 21 days after scaffold implantation, the rats were euthanized using CO_2_ inhalation. The dorsal skin was carefully resected and immediately immersed in normal saline solution. Tissue sections containing PLA and PLA-BG scaffolds were cut and fixed in 10% formalin for at least 48 h. After dehydration in 70% ethanol, the sections were embedded in paraffin wax. These sections (3 µm) were made using soft tissue microtome (Leica, Micron, Mannheim, Germany). After hematoxylin and eosin (H&E) staining, the sections were investigated for inflammatory cell infiltration and proliferation. The H&E-stained sections were observed under a polarized optical microscope (Leica DM 4500 P LED, Mannheim, Germany), and images were captured.

For statistical analysis, data were entered using Microsoft Excel and were expressed as the mean ± standard deviation. The differences in the percentage of bone formation were evaluated using the Mann–Whitney U test and compared with the Kruskal–Wallis test. A *p* value <0.005 was considered significant. SPSS statistical package version 20.0 was used for all statistical analysis. New bone formation was calculated using Image J software, and the percentage was calculated.

## 5. Conclusions

This study successfully processed and demonstrated the performance of 3D printing of a porous and biocompatible material, specifically polylactic acid–bioglass (PLA-BG) composites, for bone tissue engineering. The strategy was to take advantage of the (1) biocompatibility of the PLA and bioglass in tissue engineering, (2) potential of the TIPS method to develop PLA-BG composites with homogeneous dispersion and distribution of BG in a PLA matrix, and (3) 3D printability of PLA to develop a porous scaffold with precise control of the pore size, microstructure, scaffold geometry, and mechanical properties. PLA and PLA-BG cylindrical and disc samples with pore size distributions of 150–500 µm were 3D printed. The microscopy and tomography studies revealed well-dispersed bioglass that favorably affected the compressive strength and promoted interaction with the biological environment during the in vitro and in vivo studies. The in vitro cytotoxicity analysis of PLA-BG scaffolds using L929 mouse fibroblast cells confirmed the non-lethal interaction of the scaffold with the cells. Furthermore, morphological analysis (H&E staining) post implantation during the in vivo studies showed an elevated level of macrophages and active fibroblasts populated around the PLA-BG scaffolds that were actively involved in collagen extracellular matrix synthesis, indicating the excellent potential of these scaffolds in bone tissue engineering. Further pre-clinical studies using this scaffold to evaluate efficient bone regeneration and the biochemical- and molecular-level interactions will be performed, which will have therapeutic significance in orthopedics and dentistry.

## Figures and Tables

**Table 1:** Porosity and mechanical properties of natural bone.

	Porosity (%)	Pore Diameter (µm)	Compressive Strength (MPa)	Compressive Modulus (GPa)
Cancellous bone	75–80 50–90	300–600	2–6 2–12	0.1–0.3 0.05–0.5
Cortical bone	10	10–50	110–150 100–230	18–21 7–30

## Data Availability

Not applicable.

## Supplementary Materials

The following supporting information can be downloaded at https://www.mdpi.com/article/10.3390/molecules27217214/s1: video files (Video S1. SI_Bioglass_CubeUnif_3Dmovie.mpg; Video S2. SI_BioGlas_CubeUnif_8mu_YZ-slice_movie.mpg).
