# Supplementary material for: The Design of 3D-Printed Polylactic Acid–Bioglass Composite Scaffold: A Potential Implant Material for Bone Tissue Engineering
Source: Molecules. 2022 Oct 25;27(21):7214. doi: 10.3390/molecules27217214 (PMC9656948; doi:10.3390/molecules27217214)
Supplement: Supplementary file 1 [file molecules-27-07214-s001.zip › Electronic Supplementary Information.pdf]

Electronic Supplementary Information (ESI)

Article

Design of 3D printed polylactic acid - bioglass composite scaffold: A potential implant material for bone tissue engineering

Sahar Sultan [1], NebuThomas [2], Mekha Varghese [2], Yogesh Dalvi [3], Shilpa Joy [4], Stephen Hall [5] and Aji Mathew [1,*]

SI-1:Movie showing the 3D structure of PLA-Bioglass Uniform Cube scaffold
https://www.dropbox.com/s/szxjb4x1q3dnnb9/SI-1_BioGlas_CubeUnif_8mu_3Dmovie.mpg?dl=0

SI-2: Movie showing the slices of 3D printed PLA-Bioglass Uniform cube scaffold in the y-Z plane
https://www.dropbox.com/s/9sntm3c8qf4s4ke/SI-2_BioGlas_CubeUnif_8mu_YZ-slice_movie.mov?dl=0
